# Unexplained chest/epigastric pain in patients with normal endoscopy as a predictor for ischemic heart disease and mortality: A Danish 10-year cohort study

**DOI:** 10.1186/1471-230X-8-28

**Published:** 2008-07-15

**Authors:** Estrid Muff Munk, Bente Nørgård, Claus Dethlefsen, Hans Gregersen, Asbjørn Mohr Drewes, Peter Funch-Jensen, Henrik Toft Sørensen

**Affiliations:** 1 Department of Clinical Epidemiology, Aarhus and Aalborg Hospital, Aarhus, University Hospital, DK-8000 Aarhus C, Denmark; 2 Department of Clinical Epidemiology, Aarhus University Hospital, DK-8000 Aarhus C, and Department of Applied Research and Health Technology Assessment, Odense University Hospital, DK-5000 Odense C. Denmark; 3 Center for Cardiovascular Research, Aalborg Hospital, Aarhus University, Hospital, DK-9000 Aalborg, Denmark; 4 Center of Visceral Biomechanics and Pain, Department of Gastroenterology, Aalborg Hospital, Aarhus University Hospital, DK-9000 Aalborg, Denmark; 5 Department of Surgical Gastroenterology L, Aarhus Hospital, Aarhus University, Hospital, DK-8000 Aarhus C, Denmark

## Abstract

**Background:**

Normal upper endoscopy may be a marker of ischemic heart disease in patients with unexplained chest/epigastric pain.

**Methods:**

We examined the 10-year risk of ischemic heart disease and mortality in a cohort of 386 Danish patients with chest/epigastric pain, normal upper endoscopy, and no prior hospital discharge diagnosis of ischemic heart disease (defined as patients with unexplained chest/epigastric pain), compared with 3,793 population controls matched by age, gender, and residence. Outcome data were obtained from population-based health registries. Cox regression analysis was used to estimate the relative risk of hospitalization for ischemic heart disease and the adjusted mortality rate ratio (MRR).

**Results:**

The 10-year relative risk of hospitalization for ischemic heart disease following a normal upper endoscopy among patients with unexplained chest/epigastric pain was 1.6 (95% CI, 1.1–2.2), compared with controls. The 10-year MRR was 1.1 (95% CI, 0.9–1.5). Within the first year after the upper endoscopy the MRR was 2.4 (95% CI, 1.3–4.5). The cause-specific MRR among patients with unexplained chest/epigastric pain compared with controls was up to threefold higher for deaths related to alcohol dependence, pneumonia, and lung cancer.

**Conclusion:**

Unexplained chest/epigastric pain in patients with normal endoscopy is a strong marker for ischemic heart disease and increased mortality.

## Background

Pain originating in the upper and lower gastrointestinal tract is complex and common in the general population [1,2]. Unexplained chest/epigastric pain (UCEP) may reflect either undiagnosed thoracic or abdominal organic diseases or upper functional gastrointestinal disorders (FGIDs) [3,4]. Patients with chest and epigastric pain are often referred to gastroenterologists for evaluation of possible organic or functional causes [5,6].

In two recent cohort studies, we suggested that UCEP might reflect early symptoms of gastrointestinal cancer [7], pancreatitis, and gallstone [8]. Whether UCEP may also be a marker of increased risk of death or ischemic heart disease (IHD) remains unclear. The evidence primarily consists of small case series [9-12] and a few follow-up studies with inconsistent results and significant limitations [13-16]. Half of the studies failed to include a control group, and UCEP was defined in most cases on the basis of only chest pain and a normal coronary angiography [9-12]. More importantly, none of the studies excluded patients whose pain could have been caused by an underlying gastrointestinal disease, such as peptic ulcer or gastro-esophageal reflux disease (GERD) [9-16]. Thus, it is uncertain whether all study subjects had truly unexplained pain.

We conducted a follow-up study in Denmark to examine hospitalization for IHD, all-cause mortality, and cause-specific mortality in UCEP patients compared with population controls (using the same study population of patients as in our two previous prognostic studies on UCEP patients [7,8]).

## Methods

### Study design and population

This 10-year follow-up study was conducted at the Aarhus University Hospital in Aarhus County, Denmark. The county has a population of approximately 650,000 (12% of the Danish population). The Aarhus University Hospital has the county's largest departments of gastroenterology and surgery, and most upper endoscopies in the county are performed there. We obtained data on all patients who underwent upper endoscopy at the hospital between January 1, 1992 and December 31, 1993, with a 10-year follow-up period extending until December 31, 2003.

### Linkage between registries

Records in all registries used in this study contain the Danish Civil Registration System's unique 10-digit civil registration number, which is assigned to all Danish citizens at birth [17]. Use of the civil registration number allows valid linkage between registries.

### UCEP patients

The Aarhus University Hospital Endoscopy Registry contains both paper files and electronic medical records for all patients who underwent upper endoscopy since 1976. Since 1977 the electronic record has been maintained by the Hospital Administrative Patient Registry. Each record includes information on the patient's civil registration number, dates of admission and discharge, date and type of procedures performed, and diagnoses coded by physicians according to the International Classification of Diseases (ICD). ICD-8 codes were used in 1977–1993, and ICD-10 codes thereafter (ICD-9 was never used in Denmark) [18]. Hard-copy medical records consist of referral notes (nearly 90% of the patients are referred from general practitioners as outpatients) and endoscopy records written by the physicians who performed the procedures. The latter include information on presenting symptoms (indication for the procedure), diagnoses made during the endoscopy, biopsies taken, and description of subsequent pathological findings. This information is both standardized by means of a checklist and described in free text. General practitioners' referral notes are not standardized and mainly include information on the patients' history and symptoms. We ascertained patients' symptoms both from the endoscopy records and from the referral notes, and for the majority of patients the descriptions of the presenting symptoms from the two sources were in agreement. One of the study physicians (EMM) coded and entered data from the hard-copy medical records into an electronic research database.

The physicians who performed the upper endoscopy did not take part in the evaluation of the data of the study, selection of patients for the study, study analyses, or interpretation of the results in the study.

During the recruitment period we identified 1,799 patients with a first-time normal upper endoscopy. These patients were classified into four groups according to their symptoms: (a) only chest/epigastric pain, (b) reflux-like symptoms (*e.g.*, heartburn and/or acid reflux), (c) neither chest/epigastric pain nor reflux-like symptoms, and (d) both chest/epigastric pain and reflux-like symptoms. The subcohort of interest was comprised of the first group: 410 (23%) patients with *only *chest/epigastric pain and a first-time normal upper endoscopy. Thus, we excluded patients with symptoms such as specified/unspecified dyspepsia, heartburn and/or acid reflux or with other symptoms listed in the endoscopy record and in the referral note. Our study subcohort of patients with *only *chest/epigastric pain and a first-time normal upper endoscopy was defined and chosen *a priori *[7,8].

Through linkage to the nationwide Danish Hospital Discharge Registry (HDR), we identified patients with discharge diagnoses of ischemic heart disease (IHD) (myocardial infarction, angina, and/or heart failure [19]) prior to the date of upper endoscopy, coded according to the ICD diagnoses in Appendix 1. The HDR, established in 1977, electronically tracks all non-psychiatric hospitalizations throughout Denmark, including dates of admission and discharge, procedures performed, and up to 20 discharge diagnoses coded by medical doctors at the time of discharge. Data also on out-patients were included from 1995. We excluded 24 patients with a discharge diagnosis of IHD prior to the date of upper endoscopy. The remaining 386 patients comprised the study cohort of UCEP patients, who may resemble patients with upper FGIDs [20,21]. The study cohort was identical to the study cohort used in two recently published studies on other prognostic outcomes among UCEP patients [7,8].

### Population controls

For each UCEP patient, controls residing in Aarhus County were identified from the Civil Registration System and matched by age and gender (N = 4,100). The controls were selected on the date of the corresponding patient's first-time normal upper endoscopy (the index date). Ten controls per UCEP patient were randomly chosen to achieve statistical precision [22]. On the basis of information from HDR, 67 controls with discharge diagnoses of IHD prior to the index date were excluded. The remaining 3,793 controls were included in the analyses [7,8].

### Risk of IHD and mortality

Data on hospitalizations for IHD (defined as a discharge diagnosis of myocardial infarction, angina and/or heart failure) during the 10 years of follow up were obtained from the HDR. Mortality was ascertained from the Civil Registration System, which tracks Danish citizens' births, deaths, and migrations. In addition, death certificates, available from the Danish Causes of Deaths Registry, provided information on cause-specific mortality up to December 31, 2003. Since 1970, death certificates have included information on cause and manner of death (natural death, accident, suicide, or unknown) for 100% of deceased Danish residents. Because few patients and controls died of unnatural causes, we did not consider manner of death in our analyses.

In our cohort of UCEP patients, we focused on the seven most common causes of deaths occurring after the date of normal upper endoscopy: IHD, pneumonia, stroke, arteriosclerosis (in the absence of IHD or stroke), lung cancer, alcohol dependence, and chronic obstructive pulmonary disease.

### Confounding factors

HDR data were used to compute a comorbidity index score – the Charlson Index [23,24] – for each UCEP patient and control [7,8]. The Charlson Index, covering 19 major disease categories weighted according to their prognostic impact on patient survival, has been adapted for use with hospital discharge registry data. We computed the Index based on diagnoses recorded during all previous hospitalizations since 1977. We used discharge diagnoses of alcohol- and smoking-related diseases as proxies for alcohol abuse and tobacco smoking (ICD codes provided in Appendix 1) [7,8]. Alcohol- and smoking-related diagnoses were excluded from the Index to reduce the risk of residual confounding from these diseases. Three index levels were defined to capture increasing degrees of comorbidity: no comorbidity (Charlson Index 0), comorbidity level 1 (Charlson Index 1–2), and comorbidity level 2 (Charlson Index > 2) [7,8].

### Statistical analysis

Demographic and clinical variables such as gender, age, presence of alcohol- and smoking-related diseases, level of comorbidity, subsequent discharge diagnosis of IHD, overall mortality, and cause-specific mortality were presented as proportions or means, as appropriate.

Follow up began on the date of normal upper endoscopy or the corresponding index date for controls, and ended on the date of initial diagnosis of IHD, the date of death, the date of emigration, or at the end of the study period on December 31, 2003, whichever came first.

We constructed Kaplan-Meier survival curves and used life table techniques to estimate the risk of hospitalization for IHD and death and to summarize risk over time [7,8]. Cox regression was used to calculate the incidence rate ratio as an estimate of the relative risk and associated 95% confidence interval (CI) of hospitalization for IHD among UCEP patients compared to that for controls, while adjusting for alcohol- and smoking-related diseases and level of comorbidity [7,8]. Cox regression was also used to estimate the mortality rate ratio (MRR) and associated 95% CI for UCEP patients, relative to controls, while adjusting for alcohol- and smoking-related diseases and level of comorbidity. Similarly, Cox regression was used to estimate the MRR for cause-specific deaths. All-cause MRRs also were calculated after <1 year, 1–2 years, 3–4 years, and ≥ 5 years of follow up. For pneumonia, cause-specific MRRs were estimated for the following time periods: within <7 days, 7–31 days, and ≥31 days after the index date.

Separate analyses were performed for each type of IHD (myocardial infarction, angina, and heart failure) and stratified by time elapsed since the index date (<1 year, 1–2 years, 3–4 years, and ≥ 5 years).

Proportional hazards assumptions for the models within time periods were assessed graphically and found to be adequate. Analyses were performed using STATA version 9.1 SE (StataCorp, College Station, Texas, USA). The study was approved by the Danish Data Protection Agency (# 2001-41-1590).

## Results

Table 1 presents selected characteristics of the UCEP patients and the matched population controls. Compared with the population controls, UCEP patients had a higher prevalence of subsequent hospitalization for IHD (11% vs. 6%), were more likely to have comorbidity scores in Charlson Index category 1–2 (12% vs. 7%), and more likely to have alcohol-related diseases (8% vs. 2%). There was a slight difference between the two groups in ten-year all-cause mortality (16% of UCEP patients vs. 13% of controls). Except for deaths from alcohol dependence (1% vs. 0.1%) and pneumonia (3% vs. 1%), we found no difference in proportions of cause-specific deaths between the two groups.

**Table 1:** Characteristics of patients with unexplained chest/epigastric pain (UCEP) and matched population controls.

	UCEP patients* (N = 386)	Population controls** (N = 3,793)
Age, n (%)		
≤ 39 years	155 (40)	1,553 (41)
40–56 years	114 (30)	1,100 (29)
≥ 57 years	117 (30)	1,140 (30)
Mean age (years)	46.4	46.1
Median age (years)	44	44
Gender, n (%)		
Female	222 (58)	2,195 (58)
Male	164 (42)	1,598 (42)
Comorbidity level, n (%)†		
No comorbidity	337 (87)	3,473 (92)
Comorbidity level 1–2	45 (12)	286 (7)
Comorbidity level > 2	4 (1)	34 (1)
Discharge diagnoses, n (%)		
Alcohol-related diseases	29 (8)	79 (2)
Smoking-related diseases	19 (5)	133 (4)
Patients who developed ischemic heart disease, n (%)	39 (11)	241 (6)
10-year risk of ischemic heart disease, %	11	6
Deaths, total, n (%)	62 (16)	508 (13)
Death from ischemic heart disease#	8 (2)	76 (2)
Death from stroke	4 (1)	34 (1)
Death from arteriosclerosis (not ischemic heart disease, not stroke)	4 (1)	51 (1)
Death from pneumonia	12 (3)	41 (1)
Death from lung cancer	5 (1)	27 (1)
Death from alcohol dependence	3 (1)	5 (0.1)
Death from chronic obstructive pulmonary disease	3 (1)	38 (1)
Death from other causes	21 (5)	217 (6)
10-year mortality, %	16	13

*Normal upper endoscopy and no discharge diagnoses of ischemic heart disease before study entry.

** No discharge diagnoses of ischemic heart disease before study entry.

† No comorbidity (Charlson Index 0), comorbidity level 1 (Charlson Index 1–2). and comorbidity. level 2 (Charlson Index > 2).

# Myocardial infarction, angina, and/or heart failure.

### Risk of hospitalization for IHD

Compared with population controls, the crude relative risk of hospitalization for IHD among UCEP patients was 1.7 (95% CI, 1.2–2.4) (Table 2). Adjustment for alcohol- and smoking-related diseases and for level of comorbidity did not change the estimate. The relative risk of hospitalization for IHD within <1 year, 1–2 years, 3–4 years, and ≥ 5 years following upper endoscopy remained consistently elevated (Table 2). By type of IHD, the adjusted relative risk was 1.4 (95% CI, 0.8–2.4) for myocardial infarction, 1.9 (95% CI, 1.2–3.0) for angina, and 1.7 (95% CI, 1.0–2.9) for heart failure. The adjusted relative risk for angina and heart failure was highest in the first and the second years following upper endoscopy [4.6 (95% CI, 1.1–18.4) and 5.6 (95% CI, 1.3–23.4), respectively]. For myocardial infarction, the relative risk was the highest ≥ 5 years following upper endoscopy [2.0 (95% CI, 0.9–4.3)].

**Table 2:** Risk of hospitalization for ischemic heart disease in patients with unexplained chest/epigastric pain patients (UCEP).

	UCEP patients* (N = 386), n (%)	Population controls** (N = 3,793), n (%)	Crude relative risk	Adjusted relative risk‡
Ischemic heart disease: myocardial infarction, angina, and/or heart failure	39 (11)	241 (6)	1.7 (1.2-2.4)	1.6 (1.1-2.2)
Time of diagnosis of ischemic heart disease after upper endoscopy				
<1 year	5 (1.3)	24 (0.6)	2.1 (0.8–5.4)	1.9 (0.7–5.0)
1–2 years	5 (1.3)	19 (0.5)	2.7 (1.0–7.2)	2.5 (0.9–6.7)
3–4 years	8 (2.1)	57 (1.5)	1.5 (0.7–3.1)	1.4 (0.6–2.8)
≥ 5 years	21 (5.4)	141 (3.7)	1.6 (1.0–2.5)	1.5 (0.9–2.3)

Crude and adjusted relative risks and 95% confidence intervals for hospitalization for ischemic heart disease, overall, and by time since the index date in UCEP patients compared with population controls.

* Normal upper endoscopy and no discharge diagnoses of ischemic heart disease before study entry.

** No discharge diagnoses of ischemic heart disease before study entry.

‡ Adjusted for alcohol- and smoking related diseases, and Charlson comorbidity index: no comorbidity (Charlson Index 0), comorbidity level 1 (Charlson Index 1–2) and comorbidity level 2 (Charlson Index > 2).

### Mortality

**Figure 1:**
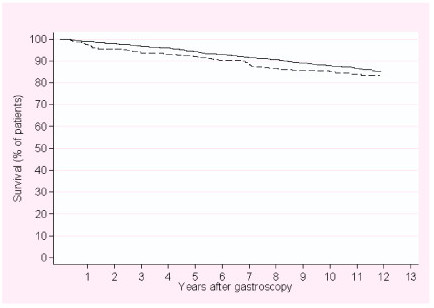
**Kaplan-Meier curves for patients with unexplained chest/epigastric pain (UCEP) (N = 386) and population controls (N = 3,793)**. --------- UCEP patients. ______ population controls.

Survival curves for UCEP patients and controls are shown in Figure 1 [see additional file 1]. The crude overall MRR was 1.2 (95% CI, 1.0–1.6) (Table 3). The estimate remained unchanged after adjustment for alcohol- and smoking-related diseases and level of comorbidity. The adjusted overall MRRs for UCEP patients within <1 year and 1–2 years after upper endoscopy were 2.4 (95% CI, 1.3–4.5) and 1.7 (95% CI, 0.8–3.9), respectively (Table 3). Thereafter, mortality among UCEP patients was comparable to that of controls (Table 3).

**Table 3:** Mortality in patients with unexplained chest/epigastric pain (UCEP).

	UCEP patients* (N = 386), n (%)	Population controls** (N = 3,793), n (%)	Crude MRR	Adjusted MRR†
Total deaths	62 (16)	508 (13)	1.2 (1.0–1.6)	1.1 (0.9–1.5)
Time to death after upper endoscopy				
<1 year	10 (3)	40 (1)	2.7 (1.5–5.0)	2.4 (1.3–4.5)
1–2 years	14 (4)	83 (2)	1.9 (0.9–4.3)	1.7 (0.8–3.9)
3–4 years	6 (2)	93 (2)	1.0 (0.6–1.8)	0.9 (0.6–1.6)
≥ 5 years	32 (8)	292 (8)	1.0 (0.7–1.5)	0.9 (0.6–1.4)
Cause-specific deaths				
Deaths from ischemic heart disease‡	8 (2)	76 (2)	1.1 (0.5–2.2)	1.1 (0.5–2.2)
Deaths from arteriosclerosis (not ischemic heart disease, not stroke)	4 (1)	51 (1)	0.8 (0.3–2.1)	0.7 (0.3–2.1)
Deaths from pneumonia	12 (3)	41 (1)	2.8 (1.5–5.4)	2.7 (1.4–5.2)
Deaths from stroke	4 (1)	34 (1)	1.1 (0.4–3.1)	1.1 (0.4–3.2)
Deaths from lung cancer	5 (1)	27 (1)	1.9 (0.7–4.9)	1.7 (0.6–4.4)
Deaths from alcohol dependence§	3 (1)	5 (0.1)	3.4 (0.7–16.8)	1.5 (0.3–8.2)
Deaths from chronic obstructive pulmonary disease¶	3 (1)	38 (1)	0.7 (0.2–2.4)	0.8 (0.2–2.5)

Crude and adjusted mortality rate ratios (MRR) and 95% confidence intervals for patients with unexplained chest/epigastric pain (UCEP) compared with population controls, overall, time-dependent, and by cause of death.

* Normal upper endoscopy and no discharge diagnoses of ischemic heart disease before study entry.

** No discharge diagnoses of ischemic heart disease before study entry.

† Adjusted for alcohol- and smoking-related diseases, and Charlson comorbidity Index: no comorbidity (Charlson Index 0), comorbidity level 1 (Charlson. Index 1–2) and comorbidity level 2 (Charlson Index > 2).

‡ Myocardial infarction, angina, and/or heart failure.

§ Not adjusted for alcohol-related diseases.

¶ Not adjusted for smoking-related disease.

Adjusted cause-specific MRRs are shown in Table 3. Elevated mortality was found for death from alcohol dependence [MRR, 1.5 (95% CI, 0.3–8.2)], pneumonia [MRR, 2.7 (95% CI, 1.4–5.2)], and lung cancer [MRR, 1.7 (95% CI, 0.6–4.4)]. Except for one case, all pneumonia deaths occurred more than 31 days after the upper endoscopy date. We found no indication of increased mortality due to IHD [MRR, 1.1 (95% CI, 0.5–2.2)].

## Discussion

The ten-year risk of hospitalization for IHD among UCEP patients was increased 1.6-fold compared with controls. The highest increase in risk was observed within the first two years following the upper endoscopy, but the risk remained elevated even after five years. All-cause mortality for UCEP patients was 16% over ten years, 1.1 times higher than the all-cause mortality in the general population. In the first year after the upper endoscopy, all-cause mortality among UCEP patients was nearly 2.5-fold higher than that of controls, but the difference faded away with time. The increased mortality among UCEP patients stemmed from alcohol dependence, pneumonia, and lung cancer, but not IHD.

The main strengths of our study are its relatively large size, a well-defined patient sample drawn from a universal-access health care system, and complete follow-up over ten years. In Denmark, nearly all patients with IHD are hospitalized during the cause of the disease (by admission to hospital either directly or through out-patient clinics), and this ensured us to registry virtually all IHD diagnoses among our cohort members. Data used to assess outcomes and potential confounders were obtained from routinely recorded discharge diagnoses and causes of death. While their quality may be variable, the positive predictive value of heart-related discharge diagnoses has been reported to be high [25]. Diagnoses recorded on death certificates are less reliable [26]. This increases the likelihood of misclassification, though most likely non-differential. To reduce variation in definition of diagnoses and symptoms, a single physician coded and entered the information from the medical records into a research database. Though some misclassification of UCEP cannot be ruled out, it is unlikely to be related to the outcomes measured.

Despite the limited level of clinical detail available in administrative data, we were able to adjust for alcohol- and smoking-related diseases and for comorbidity. However, adjusting for these potential confounders did not change our relative estimates. The Charlson Index has been shown to have high specificity, but its sensitivity is diagnosis-dependent [27], so that residual confounding by comorbidity cannot be ruled out. Further, since discharge diagnoses are proxy measurements for alcohol abuse and smoking, misclassification of these factors may have also produced residual confounding.

The UCEP patients in our study may resemble patients with upper FGIDs in characteristics such as functional chest pain of presumed esophageal origin [often termed non-cardiac chest pain (NCCP)], functional dyspepsia (epigastric pain only), or the epigastric pain syndrome [20,21]. In order to exclude patients with organic upper gastrointestinal diseases, we restricted our study to patients with a normal upper endoscopy and chest and/or epigastric pain as the sole symptom. Therefore, patients with reflux-like symptoms or dyspepsia-like symptoms described in the medical record were excluded. Likewise, we excluded UCEP patients who had received an IHD diagnosis before enrollment in the study. However, it is possible that some UCEP patients had undiagnosed IHD. Exclusion of underlying IHD is very difficult, and, as has been recently shown, even coronary angiography is not guaranteed to rule out this condition [28]. It is also possible that normal endoscopy and absence of reflux-like symptoms do not eliminate the possibility of GERD in small proportion of UCEP patients [29]. However, even the addition of pH monitoring data or documentation of response to anti-reflux therapy would not suffice to exclude GERD, because of the variable quality of such information [29]. Finally, it could be argued that patients with chest pain should be considered separately from patients with pain in the epigastrium. However, a clear differentiation of the two pain locations based on medical records was not feasible. It is well known that pain from viscera is often difficult to localize and that there is a major overlap of symptoms of pain emanating from the esophagus and related organs [1,30]. This has been also demonstrated in experimental studies [1,30-32]. Hence, the combination of pain from the chest and epigastrium appears to be a valid approach to a common clinical problem.

The increase in the risk of IHD could reflect the presence of undiagnosed IHD at the time of upper endoscopy. However, the risk remained elevated more than 5 years after the procedure. This is a strong indication that UCEP is an early marker of IHD, a finding that until now has not been reported.

The finding of increased cardiac and overall mortality among UCEP patients conforms with a previous cohort study among NCCP patients [14] and another among patients with non-ulcer dyspepsia (subgroup of FGID) [16], both using comparison groups drawn from the general population. In contrast, our results disagree with those reported in two other cohort studies among NCCP patients, both of which used asymptomatic persons as a control group [13,15]. All four cohort studies have important limitations that were avoided in our study. Three studies did not include results of upper endoscopy examinations [13-15], which could lead to inclusion of a large proportion of patients with a known underlying gastrointestinal cause of pain (up to 30% of NCCP patients [5]). In addition, the study of patients with non-ulcer dyspepsia [16] included patients with 'discomfort' and reflux-like symptoms, which are explicitly excluded from the FGIDs in the recently defined Rome III criteria in order to avoid overlap with organic disease (mainly GERD) [21]. Two studies were restricted to men aged 40–59 years [13,15]. One study examined a combined outcome (major IHD events [fatal and non-fatal]), complicating the interpretation of the result [13]. Finally, none of the studies estimated short- or long-term risks, and thus could not detect potential trends in risk.

The short-term increase in all-cause mortality among UCEP patients found in our study might be explained by the presence of severe underlying disease undiagnosed at the time of upper endoscopy, which could raise the risk of death shortly after the procedure. However, our data may indicate a truly increased risk of death from alcohol dependence, pneumonia (>31 days after procedure), and lung cancer, despite the low statistical precision of our relative mortality estimates. Pneumonia has been reported as a complication of upper endoscopy predominantly within 7 days after the procedure [33]. Therefore the risk increase observed in this study cannot be explained by the endoscopy procedure itself.

No obvious pathophysiological mechanism can explain the increase in cause-specific deaths found in our study. The findings could be due to chance or to unmeasured confounding.

## Conclusion

Our main study findings suggest that UCEP patients have substantially increased risk of hospitalization for IHD and all-cause mortality during 10-years of follow-up.

## Abbreviations

CI: Confidence interval; FGIDs: Functional gastrointestinal disorders; GERD: Gastro-esophageal reflux; HDR: Hospital Discharge Registry; ICD: International Classification of Diseases; IHD: Ischemic heart disease; MRR: Mortality rate ratio; NCCP: Non-cardiac chest pain; UCEP: Unexplained chest/epigastric pain

## Competing interests

The authors declare that they have no competing interests.

## Authors' contributions

EMM collected and analyzed the data used in the study and wrote the manuscript with contribution from BN, CD, HG, AD, PFJ, and HTS. All authors have read and approved the final manuscript.

## Appendix 1

### Discharge diagnosis according to the International Classification of Diseases (ICD)

Ischemic heart disease (IHD):

Myocardial infarction: ICD-8 code 410 and ICD-10 codes I21-23.

Angina: ICD-8 codes 411.09, 411.99 and ICD-10 code I20.

Heart failure: ICD-8 codes 402.99, 403.99, 425.99, 427.09, 427.19 and ICD-10 codes I13.0, I25.5, I42.0, I42.6-9, I50.0, I50.1, I50.9.

Pneumonia: ICD-8 code 486 and ICD-10 codes J18.0, J18.9.

Arteriosclerosis: ICD-8 code 412.9 and ICD-10 codes I25.1, I70.9.

Stroke: ICD-10 codes I61.9, I64.9, I69.4.

Lung cancer: ICD-8 code 162.1 and ICD-10 code C34.9.

Alcohol dependence: ICD-10 code F10.2.

Chronic obstructive pulmonary disease: ICD-10 codes J42.9, J43.9, J44.8, J44.9, I27.9.

Alcohol-related diseases: ICD-8 codes 303.09, 303.19, 303.20, 303.28, 303.29, 303.90, 303.99, 979, 980, 570.0, 570.9, 571, 571.09, 571.10, 573.00, 573.01, 577.10 and ICD-10 codes F10.0-9, K70.0-9, K71.1-2, K86.0-9, Z72.1, R78.0, T51.

Smoking-related diseases: ICD-8 codes 491, 492 and ICD-10 codes J40-44, J98.2, J98.3.

## Pre-publication history

The pre-publication history for this paper can be accessed here:



## Supplementary Material

Additional file 1 Table 1: Quality, psychosocial variables, participants and settings of the included reviews. This table summarises the 31 psychosocial risk factor reviews identified through the literature search. Click here for file
