# Supplementary material for: Unexplained chest/epigastric pain in patients with normal endoscopy as a predictor for ischemic heart disease and mortality: A Danish 10-year cohort study
Source: BMC Gastroenterol. 2008 Jul 15;8:28. doi: 10.1186/1471-230X-8-28 (PMC2490769; doi:10.1186/1471-230X-8-28)
Supplement: Additional file 1 — Table 1: Quality, psychosocial variables, participants and settings of the included reviews. This table summarises the 31 psychosocial risk factor reviews identified through the literature search. [file 1471-230X-8-28-S1.doc]

Figure legends

*Title of Figure 1:*

Figure 1. **Kaplan-Meier curves for patients with unexplained chest/epigastric pain (UCEP) (N=386) and population controls (N=3,793)**.

*Explanatory notes below Figure 1:*

--------- UCEP patients

______ population controls
